# The Development of an Energy Efficient Temperature Controller for Residential Use and Its Generalization Based on LSTM

**DOI:** 10.3390/s23010453

**Published:** 2023-01-01

**Authors:** Tudor George Alexandru, Adriana Alexandru, Florin Dumitru Popescu, Andrei Andraș

**Affiliations:** 1 Faculty of Industrial Engineering and Robotics, University Politehnica of Bucharest, 060042 Bucharest, Romania; 2 National Institute for Research and Development in Informatics, 011455 Bucharest, Romania; 3 Faculty of Electrical Engineering, Electronics and Information Technology, Valahia University of Targoviste, 130004 Targoviste, Romania; 4 Faculty of Mechanical and Electrical Engineering, University of Petroșani, 332009 Petrosani, Romania

**Keywords:** control, acquisition, simulation, LSTM, efficiency, PID controller

## Abstract

Thermostats operate alongside intelligent home automation systems for ensuring both the comfort of the occupants as well as the responsible use of energy. The effectiveness of such solutions relies on the ability of the adopted control methodology to respond to changes in the surrounding environment. In this regard, process disturbances such as severe wind or fluctuating ambient temperatures must be taken into account. The present paper proposes a new approach for estimating the heat transfer of residential buildings by employing a lumped parameter thermal analysis model. Various control strategies are adopted and tuned into a virtual environment. The knowledge gained is generalized by means of a long short-term memory (LSTM) neural network. Laboratory scale experiments are provided to prove the given concepts. The results achieved highlight the efficiency of the implemented temperature controller in terms of overshoot and energy consumption.

## 1. Introduction

In studies and reports dealing with the energy consumption of buildings, it was determined that an important percentage of the world energy consumption and carbon dioxide (CO_2_) emissions is linked to the residential sector [1,2,3]. Heating, ventilation and air conditioning systems (HVAC) are responsible for the vast majority of the energy usage in dwellings [4,5,6]. To achieve energy savings, it is necessary to optimize the cooling or heating process in the inside environment by regulating the heat transfer in such a way that a subtle balance is achieved between energy consumption and the comfort of the occupants. Thus, the development of sustainable HVAC systems for new buildings, and their renovation with energy saving in mind for older buildings, represents a viable means of mitigate climate change and cutting carbon emissions in this sector [7,8,9].

Intelligent (smart) home automation systems for temperature control which increase comfort and decrease the energy consumption play an important role in the above desiderata. Modeling the dynamics of room temperature is the first step in the development of temperature controllers. Afram [10] and Rao [11] classify thermal modeling approaches into three types: the white-box (or physics based) approach, the black-box (or data driven) approach, and the grey-box (or hybrid) approach.

The first modeling type [12] is based on the detailed knowledge of the process and its physics, (i.e., energy balance, thermodynamic equations, laws of conservation, etc.) and the thermal model is mathematically detailed many times by employing dedicated simulation software such as Transys [13,14], EnergyPlus [15,16], COMSOL [17,18], or Modelica [19,20].

The second modeling type does not involve any specific physical or mathematical description of the process, but rather uses data measured over time. It applies methods such as neural networks [21,22,23], nonlinear autoregressive models [24,25,26,27], or fuzzy networks [28,29] to approximate the output and model the thermal phenomena.

Finally, the third type of modeling, the grey-box approach, is a combination of the first two, benefiting from both the data-driven and physics-based models as it builds the model structure using physics-based methods and estimates the parameters of the model using performance data of the systems. This approach provides better generalization capabilities than the data-driven models and has superior accuracy to the physics-based models.

Popular grey-box models can be described as the equivalent resistor capacitor (RC) networks [30,31,32] or through machine learning, including long short-term memory models [33,34,35]. Nowadays, to simplify the model description, grey-box approaches make use of lumped parameter modeling techniques, where several elements of a certain process are “lumped” into a unique node in order to simplify the model computations. This term was originally coined in the 1970s, and ever since, a plethora of applications of the lumped parameter model [36,37,38,39,40,41,42] applied to the representation of parts of the heating controls or systems in thermal simulations have emerged.

Based on the grey-box modeling principles, our paper proposes a new approach for the estimation of heat transfer in residential buildings, using a lumped parameter thermal analysis model. The scope of our research is to develop a temperature controller and its generalization using machine learning by means of a LSTM neural network. The original contribution consists of the actual model development, the analysis of the disturbances on the lumped conductance and capacitance of the system, and the control variable generalization based on the recorded weather data.

Future work will include correlating the data processed from the smart home systems, such as the occupants’ behavior patterns/templates and the controller model, in order to study their influence on the artificial intelligence algorithm.

The paper is organized as follows: Section 1 Reviews Literature On Modeling The room temperature control, with emphasis on grey-box methods, where our approach also fits; Section 2 introduces the theoretical considerations regarding heat transfer in residential buildings; and Section 3 describes the proposed approach, at first schematically and then by detailing the methodology step-by-step. In the first stage, a laboratory scale experimental setup is developed, including a thermostat circuit. A simulation model is proposed for recreating the heat transfer that occurs in the interior space of the enclosure. The lumped conductance and capacitance of the fluid domain are parameterized based on experiments that were carried out in a disturbance free environment. In the next stage, the thermal analysis module is coupled with a numerical computing environment for developing and tuning a proportional-integral-derivative (PID) temperature controller. Benchmarking of the resulting model was completed during November 2021. The experiments took place in the interior and exterior locations of the Laboratory of Prototypes, Building CF from the University Politehnica of Bucharest, Romania. A wide range of process disturbances were identified and correlated with the weather conditions of that month. Based on these findings, this paper proposes an improved control methodology. Its generalization is carried out using an LSTM neural network for facilitating its future use as part of smart home automation systems.

In the final section, discussion of the results takes place and concluding remarks are drawn.

## 2. Theoretical Aspects of Heat Transfer Inside Residential Buildings

Generally speaking, the thermal exchange between two systems is called heat transfer, occurring from the hot to the cold system. In the case of buildings, heat transfer inside them is important from the design phase, starting from choice of building material and the passive and active HVAC systems required to assure an optimal thermal condition with minimum resource consumption. Heat transfer happens by various mechanisms such as conduction, convection, and radiation of phase change, and the thermal behavior of a building or room is governed by a function of the dynamic relationship between these mechanisms. Usually, heat is transferred within solids by conduction, in fluids by conduction and convection, and in open space by radiation. In practice, these types of heat transfer may occur together.

In the case of rooms, heat energy is transferred by conduction, convection, and radiation. Conduction describes heat transfer through walls, the floor, ceiling, and furniture, convection describes heat transfer in the internal air inside the room, and radiation describes the heat coming from the outside through the windows but mostly from heat sources (radiators, stoves, etc.) to objects in the room.

In the case of radiation from the heat sources in a room, if the environment has objects inside that are both transparent (windows, doors, glass, etc.) and opaque (furniture, appliances, walls, etc.), surface-to-surface type radiation is assumed. Thus, the radiative heat flux can be defined for both opaque and transparent surfaces.

### 2.1. Radiative Heat Flux in the Case of Opaque Surfaces

**Figure 1:**
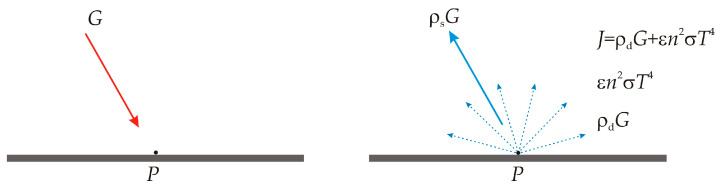
Representation of irradiation *G* and radiosity *J*.

Consider a body with an opaque surface which transmits no radiation. For its surface with absorptivity *α*, emissivity ε, diffuse reflectivity ρ*_d_*, specular reflectivity ρ*_s_*, refractive index *n*, and temperature *T*, if we consider a point **P** on that surface, there is an incoming radiative flux *G* called irradiation and an outgoing radiative flux *J* called radiosity [43], as represented in Figure 1. 

Radiosity is defined as the quantity of emitted radiation summed with the diffusely reflected radiation:

(1) $$J=\varepsilon \cdot e_{b}(T)+\rho_{d}\cdot G$$

where $e_{b}(T)$ is the total radiated power across all wavelengths. Based on the Stefan-Boltzmann law, this is dependent on the fourth power of the temperature *T*:

(2) $$e_{b}(T)=T^{4}n^{2}\sigma$$

where *n* is the refractive index, and *σ* = 5.67 · 10^−8^ W/m^2^K^4^ is the Stefan–Boltzmann constant.

Then, the net inward radiative heat flux *Q* results as the difference between irradiation and outgoing radiation (i.e., specular reflected radiation summed with radiosity):

(3) $$Q=G-(\rho_{s}G+J)$$

which can be also expressed as:

(4) $$Q=(1-\rho_{s})G-J.$$

The radiosity *J* can be removed from equations 1 to 4, thus a general expression of the net inward heat flux can be written based on *G* and *T* for opaque bodies, as:

(5) $$Q=[1-(\rho_{d}+\rho_{s})]G-\varepsilon \cdot e_{b}(T).$$

Since most opaque bodies behave as ideal gray bodies, the emissivity and absorptivity are equal, and then the reflectivity $\left(\rho_{d}+\rho_{s}\right)$ can be derived from the equation:

(6) $$\varepsilon =\alpha =1-(\rho_{d}+\rho_{s}).$$

So, for the ideal gray bodies, the inward radiative heat flux *q* can be expressed as:

(7) $$Q=\varepsilon (G-e_{b}(T)).$$

### 2.2. Radiative Heat Flux in the Case of Semi-Transparent Surfaces

**Figure 2:**
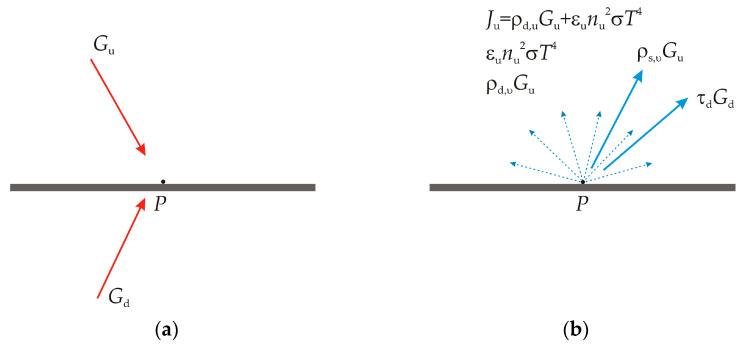
(**a**) Representation of upper side and lower side incoming radiation *G_u_, G_d_* and (**b**) upper side outgoing radiosity *J_u_*.

Consider a body with a semi-transparent surface which transmits some radiation. For such a body, we consider a point **P** on this surface, with upper side emissivity ε*_u_*, diffuse reflectivity ρ*_d,u_*, specular reflectivity ρ*_s,u_*, refractive index *n_u_*, temperature *T_u_*, lower side emissivity ε*_d_*, diffuse reflectivity ρ*_d,d_*, specular reflectivity ρ*_s,d_*, refractive index *n_d_*, and temperature *T_d_* as shown in Figure 2.

For this point **P,** there is an incoming radiative flux called irradiation as shown in Figure 2a., noted with *G_u_* on the upper side and *G_d_* on the lower side, and a total diffuse outgoing radiative flux called radiosity and noted with *J_u_* on the upper side (Figure 2b) and *J_d_* on the lower side. This radiosity is the sum of emitted radiation, transmitted radiation, and diffusively reflected radiation from the other side of the semi-transparent body:

(8) $$J_{u}=\varepsilon_{u}\cdot e_{b,u}(T_{u})+\rho_{d,u}\cdot G_{u}$$

(9) $$J_{d}=\varepsilon_{d}\cdot e_{b,d}(T_{d})+\rho_{d,d}\cdot G_{d}.$$

The net inward radiative heat fluxes on the upper and lower side, *Q_u_* and *Q_d_,* respectively, are given as the difference between the irradiation and the radiosity:

(10) $$Q_{u}=(1-\rho_{s,u}-\tau_{u})G_{u}-J_{u}$$

(11) $$Q_{d}=(1-\rho_{s,d}-\tau_{d})G_{d}-J_{d}.$$

Bodies behave like ideal gray bodies, so the emissivity is equal to the absorptivity. Hence, the reflectivity ρ*_s_* can be obtained from equations:

(12) $$\varepsilon_{u}+\rho_{d,u}=1-\rho_{s,u}-\tau_{u}$$

(13) $$\varepsilon_{d}+\rho_{d,d}=1-\rho_{s,d}-\tau_{d}.$$

If *J_u_* and *J_d_* are eliminated from Equations (8) to (13), a general expression of the net inward heat fluxes can be written, based on *G_u_*, *G_d_*, *T_u_*, and *T_d_* for semi-transparent bodies:

(14) $$Q_{u}=\varepsilon_{u}(G_{u}-e_{b,u}(T_{u}))$$

(15) $$Q_{d}=\varepsilon_{d}(G_{d}-e_{b,d}(T_{d})).$$

So, for the ideal gray bodies, the inward radiative heat flux *Q* can be expressed as:

(16) $$Q=\varepsilon_{u}(G_{u}-e_{b,u}(T_{u}))+Q_{d}=\varepsilon_{d}(G_{d}-e_{b,d}(T_{d})).$$

Regardless of the transmittance of the surface, rays incident at an angle (measured between the ray and its normal to the surface) bigger than the critical angle *θ_c_* are not transmitted but have a contribution to the total reflection. As a result, a coefficient of directional transmissivity can be defined:

(17) $$\tau (\theta )=\left\{ \begin{array}{l} \tau \;for\;\theta \leq \theta_{c}\\ 0\;for\;\theta >\theta_{c} \end{array}\right..$$

Based on the equation:

(18) $$\rho_{s}(\theta )+\tau (\theta )=1-(\varepsilon +\rho_{d})$$

it can be written that:

(19) $$\rho_{s}(\theta )=\left\{ \begin{array}{l} \rho_{s}\;for\;\theta \leq \theta_{c}\\ \rho_{s}+\tau \;for\;\theta >\theta_{c} \end{array}\right..$$

**Figure 3:**
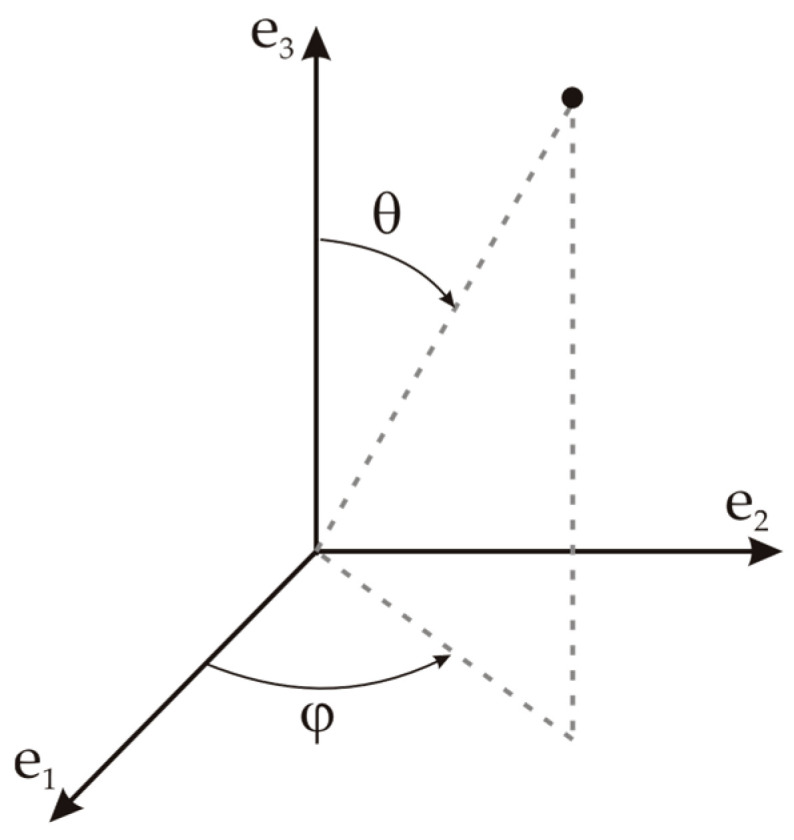
Polar and azimuth angle representation.

Surface to surface radiation modeling uses the radiosity method, where temperature and wavelength depend on surface properties. The ray-shooting approach is used to estimate emissivity, specular reflectivity, and transmissivity, which are dependent on the incident angle. Next, the azimuth and polar angles can be defined as θ∈[0, π/2] and φ∈[−π, π] as shown in Figure 3. The directional surface properties can be decomposed as sums and defined as functions of θ, φ, and also other quantities such as temperature or spatial coordinates.

Emissivity (Equation (20)), transmissivity (Equation (21)), and specular reflectivity (Equation (22)) can be expressed, respectively, as:

(20) $$\varepsilon_{tot}(\theta ,\varphi ,T,x)=f_{\varepsilon }(\theta ,\varphi )+\varepsilon (T,x)$$

(21) $$\tau_{tot}(\theta ,\varphi ,T,x)=f_{\tau }(\theta ,\varphi )+\tau (T,x)$$

(22) $$\rho_{s,tot}(\theta ,\varphi ,T,x)=1-\rho_{d}-\varepsilon_{tot}(\theta ,\varphi ,T,x)-\tau_{tot}(\theta ,\varphi ,T,x).$$

### 2.3. Evalation of the View Factor

A view factor is a measure of the influence of radiosity in a given part of a boundary on the irradiation at a different part. The total irradiation *G* at a given point is the sum of three contributing irradiations: the mutual irradiation *G_m_* that comes from other boundaries, the irradiation from external sources *G_ext_*, and the ambient irradiation *G_amb_*. It can be written as:

(23) $$G=G_{m}+G_{ext}+G_{amb}=G_{m}+G_{ext}+\varepsilon_{amb}F_{amb}e_{b}(T).$$

The radiosity *J* is a function of *G_m_*, so the radiation balance equation can be written as:

(24) $$J=\rho_{d}(G_{m}(J)+G_{ext}+G_{amb})+\varepsilon e_{b}(T).$$

**Figure 4:**
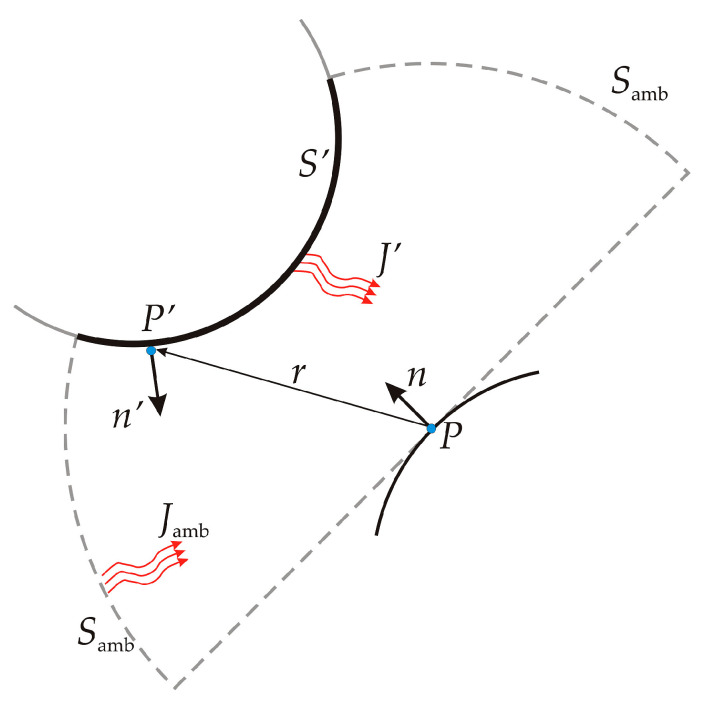
Geometry representation for surface-to-surface radiation.

In Equation (23), *F_amb_* is the ambient view factor and *G_m_* is the integral of the differential view factor on all visible points times the radiosity of the source point of the radiation. If we consider a point **P** on a surface as represented in Figure 4, it is visible by points such as **P^’^** on other surfaces such as **S^’^** and also from the ambient surroundings **S_amb_**.

If it is assumed that points on surface **S^’^** have a radiosity noted *J^’^* and that the ambient surroundings have a constant temperature noted *T_amb_*, then the mutual irradiation *G_m_* at point **P** is expressed by the integral:

(25) $$G_{m}=\int_{S^{\prime }}\frac{\left(-n^{\prime }\cdot r)(n\cdot r\right)}{\pi {\left|r\right|}^{4}}J^{\prime }ds.$$

The heat flux arriving from **P^’^** depends on the radiosity *J^’^* projected on **P**, where the projection is calculated using normal vectors **n** and **n’** and vector **r** pointing from P to P’. Similarly, the ambient view factor *F_amb_* can be determined from the integral of the surfaces **S’** and can be written as:

(26) $$F_{amb}=1-F^{\prime }=1-\int_{S^{\prime }}\frac{\left(-n^{\prime }\cdot r)(n\cdot r\right)}{\pi {\left|r\right|}^{4}}ds.$$

## 3. Materials and Methods

### 3.1. A Schematic Representation of the Proposed Approach

**Figure 5:**
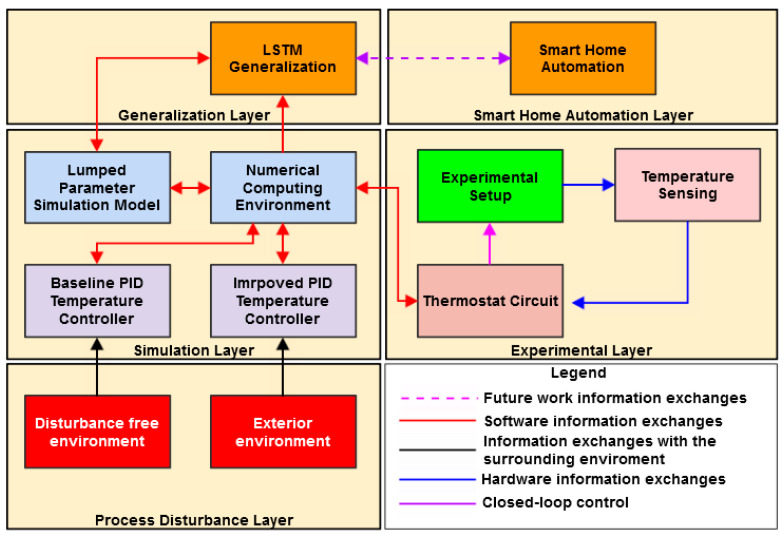
The proposed approach and its layers of abstraction.

The proposed methodology consists of five layers of abstraction that divide the problem of thermal modeling and control in distinct hardware and software practices. Figure 5 depicts a schematic representation of the approach with an emphasis on the information exchanges that take place.

A description of the abstraction layers is discussed below:

- **The experimental layer** stands at the core of the approach, comprising a laboratory scale experimental setup that is employed for capturing both the heat transfer and temperature control problems of interior spaces that are found in residential buildings. In this regard, temperature sensing hardware and a thermostat circuit are included. The interaction between the two materializes a closed-loop controller;
- **The simulation layer** includes the software that is required for modeling the plant (the combination between the experimental setup and its heating system) by means of a lumped parameter simulation model. Experimental data is used for parameterizing the conductance and capacitance of the fluid domain that is found in the interior space of the enclosure. A numeric computing environment is employed for developing and tuning a PID temperature controller. This objective is achieved at first in a disturbance free environment and then by repeating the experiments in the exterior for one month. The conductance and capacitance of the fluid domain are adjusted to account for process disturbances due to weather conditions. Thus, an improved control methodology can be achieved;
- **The process disturbance layer** materializes two physical environments: one interior environment that is characterized by no disturbing factors (i.e., temperature variations or fluctuating heat transfer due to forced convection) and the exterior environment that is subjected to process disturbances due to the weather conditions. Both environments are used for developing and tuning an adequate control methodology;
- **The generalization layer**: the knowledge gained throughout the research can be encompassed in sequential data that characterizes the response of the improved control methodology when subjected to process disturbances. Thus, a LSTM recurrent neural network can be employed to generalize the behavior of the controller for any given scenario;
- **The smart home automation layer**: machine learning is a constitutive part of smart home automation. Thus, the LSTM model developed in the previous layer can interact with such domestic systems. Furthermore, multiple features can be included in the supervised learning process for widening the sources of process disturbances.

### 3.2. The Experimental Platform

A laboratory scale experimental setup is employed for recreating the heat transfer that occurs in residential buildings. In this regard, a 300 × 300 × 250 mm enclosure is developed by employing common building materials.

The setup consists of a poplar plywood enclosure that is held together by means of 4 cornices. The exterior walls of the structure are insulated by polystyrene panels. Plexiglas windows are positioned in the centroid of two opposing walls. The heating of the internal space is achieved by employing ceramic resistors as heating elements.

**Figure 6:**
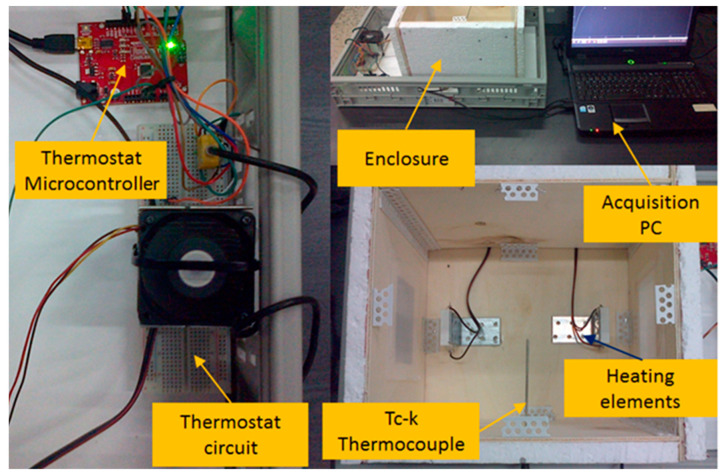
The experimental setup and its main subsystems.

As seen in Figure 6, monitoring and control of the thermostat circuit is carried out by means of a microcontroller and an acquisition PC.

**Figure 7:**
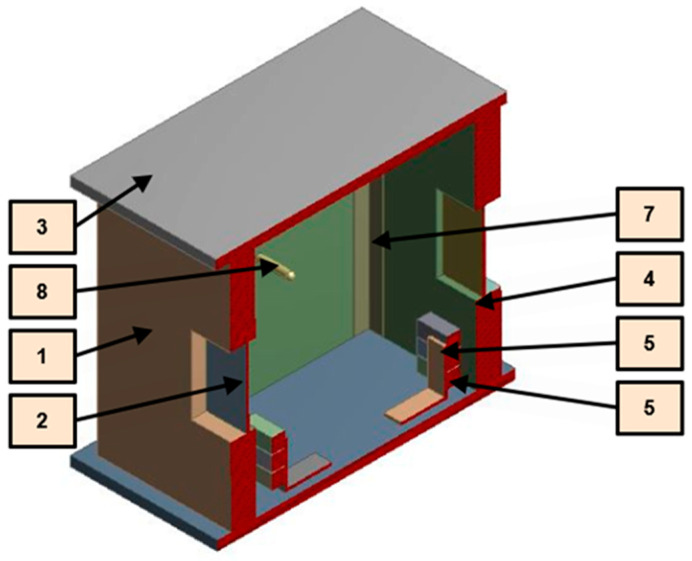
Description of the laboratory scale test platform.

The main components of the experimental setup are emphasized in Figure 7 with detailed description, name, and numbering described in Table 1.

**Figure 8:**
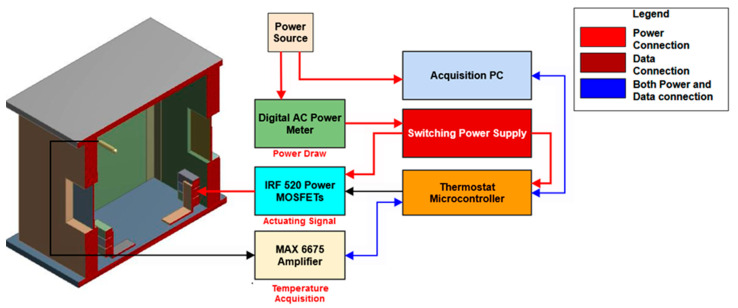
Schematic representation of the thermostat and temperature acquisition circuits.

The control and monitoring of the internal temperature of the enclosure are achieved with the support of an external circuit, which is represented schematically in Figure 8.

Temperature feedback is provided by a Tc-k thermocouple that is connected to a MAX6675 amplifier for cold junction compensation. The output signal achieved by means of the serial peripheral interface (SPI) communication protocol is fed to the digital input pins of the thermostat microcontroller (Sparkfun Red Board).

The maximum power that is dissipated by the resistors is controlled by means of a power metal–oxide–semiconductor field-effect transistor (MOSFET). An active cooling system is employed to maintain the temperature of the MOSFET below its operational limits. The actuating signal is generated by employing the pulse-width modulation (PWM) technique on the digital output pins of the microcontroller.

A switching power supply is used for powering up the thermostat and the heating elements. An AC power meter is employed for benchmarking the energy usage of the thermostat circuit.

### 3.3. The Simulation Model

A lumped parameter model is developed for capturing the thermal behavior of both fluid and solid domains of the enclosure. This objective is achieved by employing a 2D heat transfer finite element method (FEM) approach. The governing equation of heat conduction is expressed by [44]:

(27) $$\frac{\partial }{\partial x}\cdot \left(k\cdot \frac{\partial T}{\partial x}\right)+\frac{\partial }{\partial y}\cdot \left(k\cdot \frac{\partial T}{\partial y}\right)+G=0$$

where *T* represents the nodal temperatures, *k* the thermal conductivity, and *G* the internal heat generation.

Only a section of the entire structure is analyzed, comprising a planar representation of the side walls, windows, and the heating elements. The air inside the enclosure is modeled by membrane elements. Thermal contact conductance (*TCC*) is included for limiting the thermal flux that occurs at the boundary between the solid and fluid domains [45]:

(28) $$q=k_{c}\cdot \left(T_{i}-T_{j}\right)$$

where *q* represents the thermal flux, *k_e_* the thermal contact conductance, and *T_i,j_* the temperatures at the contacting nodes.

The capacitance (*C_th_*) of the fluid domain is modeled by employing a 0D thermal mass element that is characterized by the specific heat matrix [46]:

(29) $$\left[C_{e}^{t}\right]=\left[C_{th}\right]$$

where *C_th_* represents the lumped capacitance of the element.

Equations (28) and (29) are employed to overcome the unrealistic solid representation of the fluid domain.

Given the fact that thermal analysis by FEM materializes a first order system, a lumped parameter behavior can be imposed by matching the time constant and temperature gain of the system with experimentally derived values. Symmetry is used to lower the computational demands of the solving process. The vertical planes of the exterior walls materialize the cold plate. In this regard, isothermal natural convection is assumed, the value of the film coefficient being dependent on the environmental temperature.

**Figure 9:**
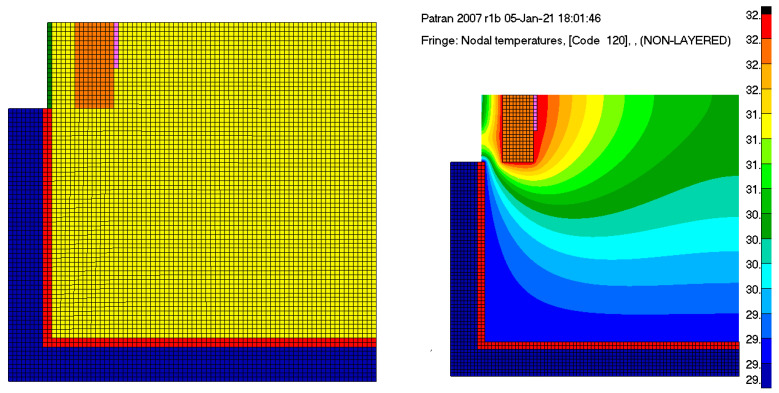
Representation of the 2D heat transfer simulation approach.

Figure 9 depicts the simulation model and an example of temperature gradients that occur at the fluid domain level when the heating elements are switched on at full power, with an ambient temperature of 18 °C.

The following software were used for completing the study:

- **MSC Patran 2007:** a general purpose pre- and postprocessor which was used in the first stage for the graphical definition of the finite elements that materialize the experimental setup. Sections of the simulation input file (i.e., the material data or the heat transfer conditions) were defined based on an automated procedure. After solving the equations, the result files were processed as tabular data and fringe plots;
- **LMS SAMCEF v15.1**: a finite element analysis solver package which includes an extended library of macro elements. Using it, lumped parameter modeling can be carried out for a wide range of thermal and mechanical problems. Another advantage of this software suite is its direct interfacing ability with numerical computing environments, such as MATLAB. Therefore, the solver can operate either standalone (when an input file is provided) or as co-simulation (when results of the analysis are used as input for the numerical computing environment or vice-versa).

### 3.4. Identifying the Lumped Parameters of the Simulation Model

**Figure 10:**
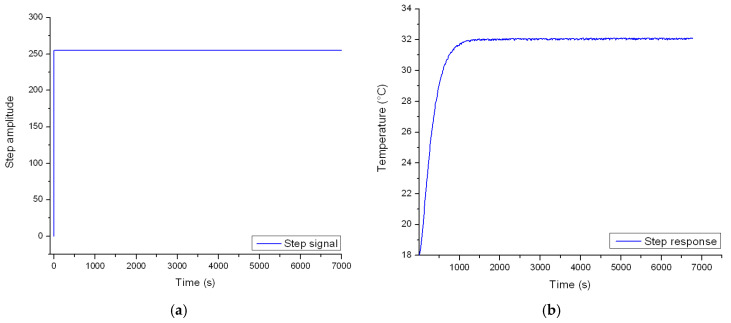
(**a**) Step input PWM signal and (**b**) output response temperature.

*TCC* and *C_th_* can only be identified by evaluating the step response of the real-world experimental setup. To this end, the PWM signal is set to its limit value. This change takes place in one second, causing the heat that is dissipated by the resistors to max out. The objective of the experiment is to evaluate the temperature of the fluid domain inside the enclosure, until an equilibrium value is reached. The study was carried out in an open space environment that has a constant ambient temperature of 18 °C throughout the test sequence. Figure 10a depicts the shape of the actuating signal while Figure 10b illustrates the time vs. temperature curve.

Afterwards, the time constant and temperature gain of the resulting curve are evaluated.

**Figure 11:**
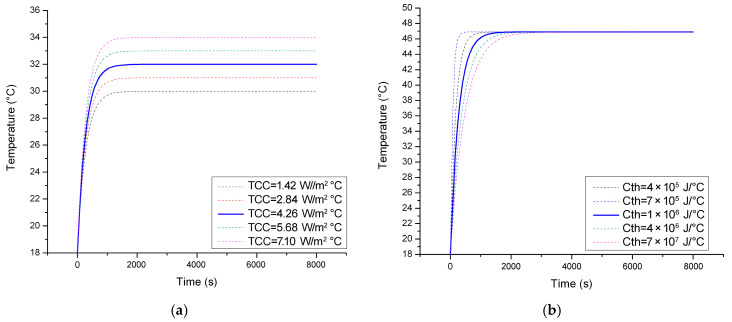
(**a**) Matching the *TCC* and (**b**) *C_th_* of the model to the behavior of the physical system.

In the next stage, the *TCC* (Figure 11a) and *C_th_* (Figure 11b) of the simulation model are decided based on a sensitivity study that highlights the relationship between the two variables and the response of the system.

Given the linear relationship between the values, the baseline *TCC* was decided as 1.04 W/m^2^·°C, while the *C_th_* that matches the time constant of the system was estimated as 1 · 10^6^ J/°C.

### 3.5. Development of a PID Thermostat

The development of the PID thermostat requires, in the first stage, coupling the lumped parameter simulation model with a numerical computing environment for developing and tuning a temperature control methodology. MATLAB Simulink was chosen as the ideal candidate. On one hand, it has the ability to exchange information with finite element solvers. On the other, its toolboxes ease the control methodology development process. Furthermore, data acquisition can be carried out with the thermostat microcontroller to implement the controller in the real-world environment.

A co-simulation approach is proposed by coupling SAMCEF Thermal with MATLAB Simulink. This objective is achieved with the support of an S-function [47]:

(30) $$F\left(x,y,t\right)=\left(\begin{array}{c} f\left(x;y,t\right)\\ g\left(y;x,t\right) \end{array}\right)=\left(\begin{array}{c} 0\\ 0 \end{array}\right)$$

where *x* represents the nodal temperature of the fluid domain from the SAMCEF environment and *y* represents the actuating signal that is generated by the closed-loop controller in MATLAB Simulink. In Equation (27), *f*(*x*; *y*, *t*) *=* 0 and *g*(*y*; *x*, *t*) = 0 are solved for the unknown *x* for a given value of *y* and *t*.

**Figure 12:**
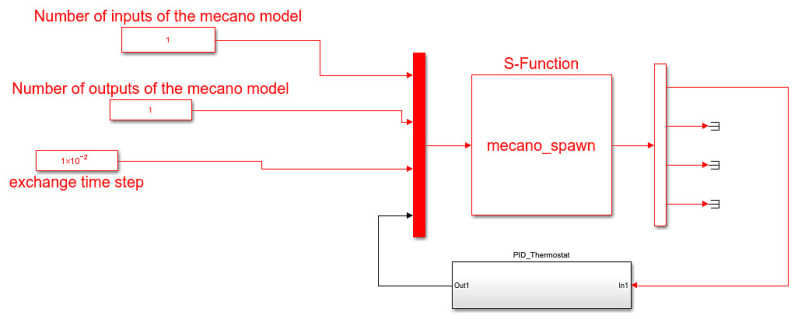
Simulink–SAMCEF co-simulation strategy.

Figure 12 depicts the schematic representation of the Simulink model used for developing and tuning the PID thermostat.

The data transfer between Simulink and SAMCEF occurs based on one input (the power dissipation at the level of the resistors) and one output (the nodal temperature of the fluid domain). For achieving convergence, the exchange time step was adjusted to 0.01 s. The PID Thermostat subsystem includes the PID controller definition in the parallel form [48]:

(31) $$C=K_{p}+\frac{K_{i}}{s}+\frac{K_{d}s}{Tf_{s}+1}$$

where *K_p_* represents the proportional gain, *K_i_* the integral gain, *K_d_* the derivative gain, and *T_f_* the first-order derivative filter time constant.

Saturation limit is imposed at the maximum output of the PWM signal. On the other hand, the clamping method is employed to avoid integral windup [49].

Tuning of the controller gains is carried out by employing the PID tuner toolbox to achieve a balance between performance and robustness [50]. For the given plant, the tuning constants were decided as: *K_p_* = 23.95, *K_i_* = 0.15, and *K_d_* = −219, with a filtered derivative coefficient of 0.009. Based on the defined gains, the controller has a rise time of 271 s, a settling time of 964 s, and an overshoot of 7.9%.

### 3.6. Ideal Environment Simulation

**Figure 13:**
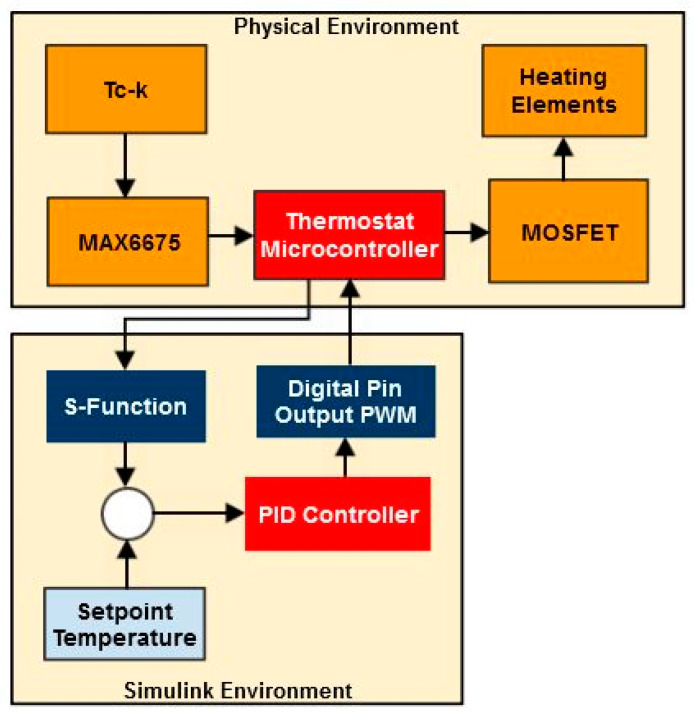
The connection between Simulink and the physical hardware.

In the next stage, the Simulink environment is set up to interactively communicate with the microcontroller of the thermostat. Thus, the output of the PID controller is converted into a PWM signal that will adjust the input voltage of the heating elements. The temperature change in the plant is measured by means of SPI data acquisition that is carried out from the MAX6675 amplifier. An S-function is defined for this purpose. Figure 13 depicts the information exchange between the physical hardware and the Simulink PID controller.

**Figure 14:**
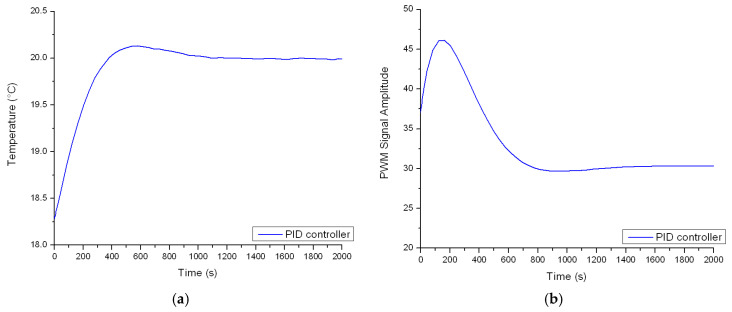
(**a**) Time vs. temperature graph and (**b**) shape of actuating signal.

The thermostat is tested for a setpoint value of 20 °C. Figure 14 emphasizes the behavior of the controller in terms of input (amplitude of the PWM signal) and output values (the temperature inside of the enclosure).

### 3.7. Simulation in the Exterior Environment

**Figure 15:**
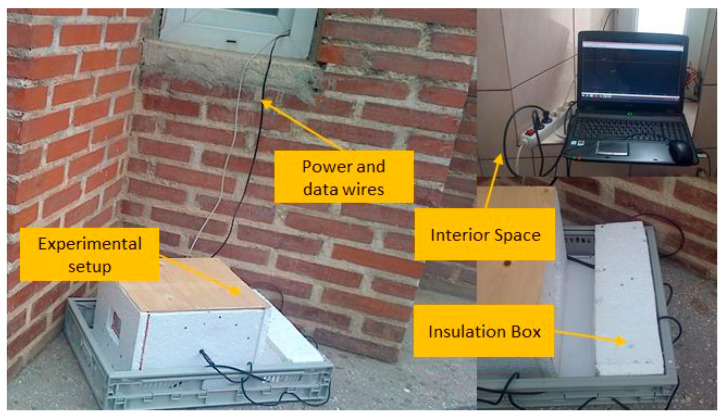
The experimental setup employed in the exterior environment.

The exterior environment of residential buildings represents a source of heat transfer disturbances for thermostats; for example, changes in the wind speed or direction cause the forced convection film coefficients of the external walls to change. On the other hand, the amount of cloud covering the sky limits or enhances the thermal gradients occurring on the exterior surface of windows due to radiation heat transfer. Therefore, the performances of the temperature controller can only be verified by testing the operation of the experimental setup in the exterior environment. To this end, the enclosure and its electronics were positioned outside of the building as shown in Figure 15.

**Figure 16:**
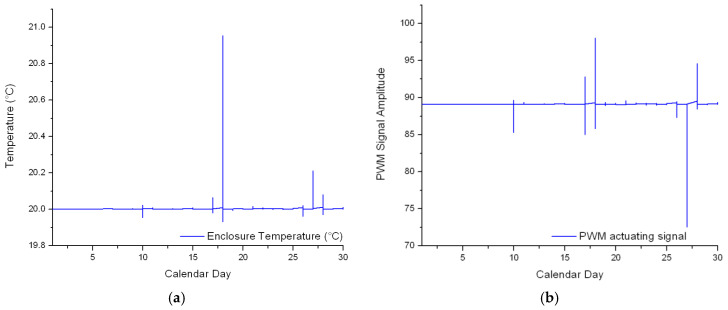
(**a**) Temperature inside the enclosure and (**b**) actuating signal of the controller.

The circuit of the thermostat was insulated to prevent its damage due to moisture or low temperatures. Moreover, the acquisition PC and the switching power supply were kept inside. Experiments were conducted between the 1 and 30 November 2021 at the Laboratory of Prototypes, Building CF from the Politehnica University of Bucharest. The set point temperature was decided as 20 °C. Figure 16 depicts the time vs. temperature graph and the shape of the PWM actuating signal for the entire simulation period.

### 3.8. Evaluation of the Process Disturbances

While the controller proved the ability to maintain the temperature inside the enclosure at 20 °C, an overshoot of +1 °C can be noticed on the 18 November. On the other hand, an undershoot value of −0.8 °C can be noticed on the 10 November. In total, the Digital AC Power Meter has recorded a 10.56 KWh power draw for the entire period, and the maximum instantaneous power draw recorded was 0.041 KW when the thermostat circuit was operating at full capacity.

These events were correlated with the weather conditions that were extracted from the Ido.ro website (Table 2) [51].

A recurring pattern can be noticed between the minimum, maximum, and average values. For example, the average temperature difference between the two days is 8.35 °C. On the other hand, the average humidity difference between the two days is 43%. Furthermore, the average wind speed difference is 12 Km/h while the average pressure difference is 3.4 mmHg.

**Figure 17:**
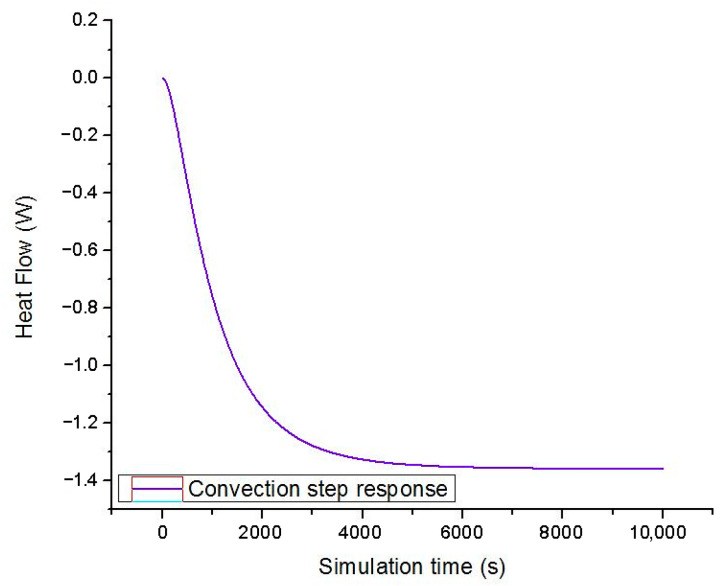
Nodal reaction heat flow of the convection boundary condition.

To quantify these process disturbances, the actuating signal derived from the controller for the 18 November is transferred to the SAMCEF simulation model. Given the fact that the environment surrounding the enclosure is a catalyst for heat removal (by natural or forced convection) or heat generation (by radiation), it is important to take such influences into account. In the ideal simulation model, heat is removed from the enclosure by isothermal natural convection. For the maximum output of the resistors, the nodal reaction occurring on the exterior walls is depicted in Figure 17.

The resulting curve can be approximated by means of a transfer function with a single pole and no zeroes by employing the system identification roolbox from MATLAB [52]:

(32) $$G\left(s\right)=\frac{-0.0013}{s+0.000094}.$$

A 95% match was achieved between the nodal reaction of the simulation model and the step response of the derived first order system.

In the next stage, the inverse of the resulting transfer function is carried out by multiplying the inverted *G*(*s*) with a fast dynamics second order transfer function:

(33) $$G{\left(s\right)}_{inverted}=\frac{1}{G\left(s\right)}\cdot \frac{1}{0.01\cdot s^{2}+0.2\cdot s+1}.$$

Thus, a correlation can be achieved between the actuating signal and the heat that is absorbed or released through the external walls of the enclosure.

**Figure 18:**
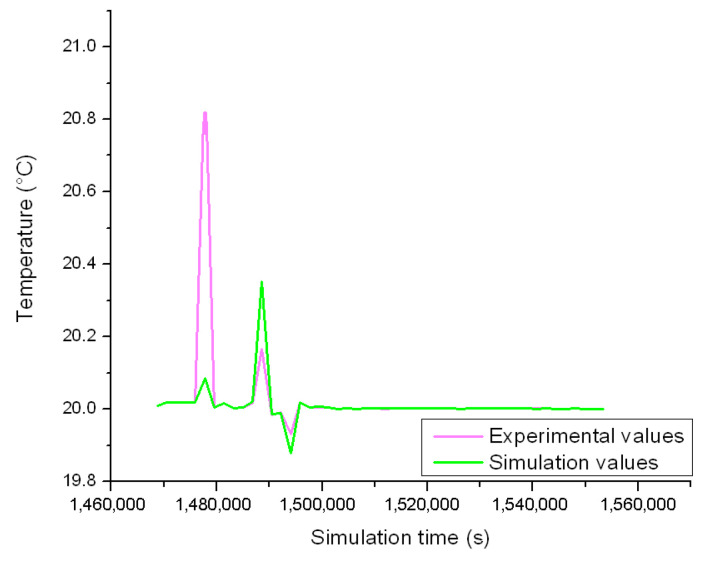
Experimental vs. simulation temperature curves for the 18 November.

In the next stage, the energy that is dissipated by the resistors is scheduled in the lumped parameter model, alongside the power that is exchanged through the external walls, to recreate the behavior of the system for the 18 November (Figure 18).

**Figure 19:**
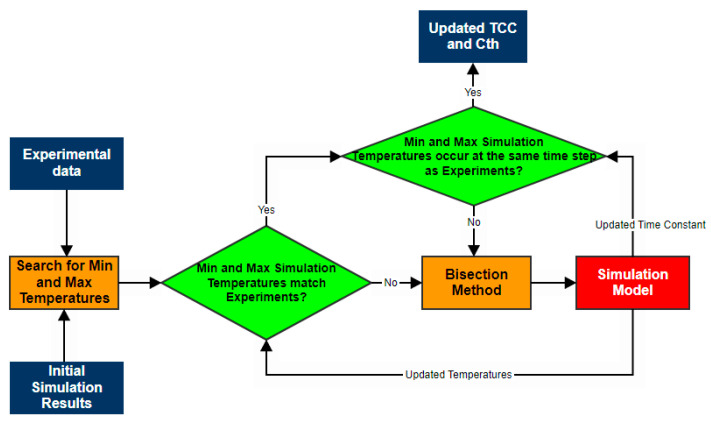
Flowchart of the algorithm employing for quantifying the process disturbances.

By comparing the experimental values with the ones achieved by simulation, the impact of the process disturbances is clearly visible in case of the minimum and maximum temperatures. A parametric study is developed to limit such error sources by employing an algorithm that is based on the bisection method (Figure 19).

In the first stage, the maximum and minimum temperatures are extracted from the initial experimental and simulation results. The bisection method is employed to update the *TCC* of the fluid domain in the simulation model if the identified values have differences between them that exceed 2%. This process is carried out until convergence is achieved. In the next stage, the same approach is employed for evaluating the time step at which the minimum and maximum temperature values occur. For the present study, corrections of −4.97% were applied to the initial conductance of the model. On the other hand, corrections of +6.23% were required for the initial capacitance of the model. Due to these changes, the behavior of the plant is different compared to the ideal environment. Thus, the controller gains require retuning. The new values are: *K_p_* = 2.04, *K_i_* = 0.001, and *K_d_* = −277.9 with a filter coefficient of 0.009.

**Figure 20:**
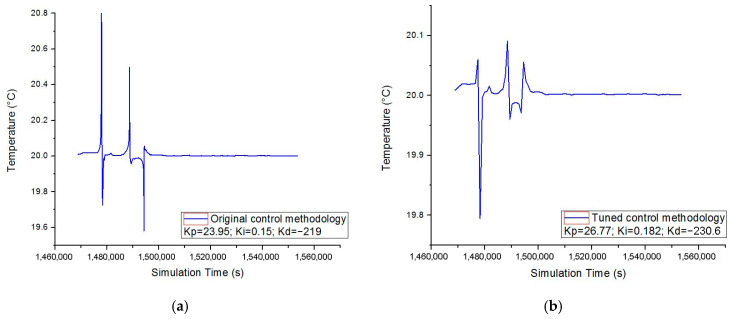
(**a**) Temperature inside the enclosure for the original control methodology and (**b**) temperature inside the enclosure for the tuned control methodology.

To estimate the energy savings due to the tuned control methodology, the amplitude of the actuating signal is correlated with the maximum instantaneous power draw from the thermostat circuit (0.041 KW from the log of the Digital AC Power Meter). In this case, the improved methodology demands 0.325 KWh while the original controller required 0.344 KWh. Thus, energy savings of 2.63% can be achieved. Furthermore, the controller managed to maintain the setpoint temperature, the maximum error being +0.1 °C instead of +0.8 °C (Figure 20).

Based on these findings, the methodology was extended for the entire month. Table 3 depicts the *TCC* and *C_th_* corrections and the tuned controller gains for a selection of days with high process disturbances.

**Figure 21:**
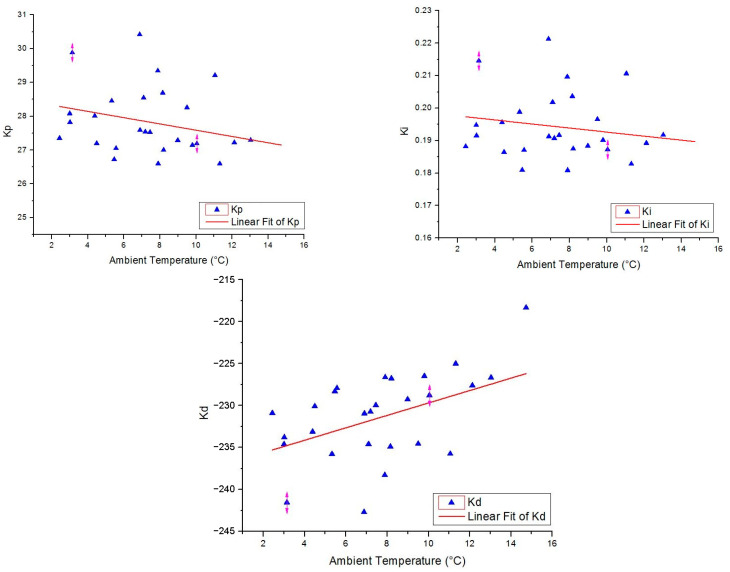
Linear relationship between the ambient temperature and the tuned controller gains.

In the next stage, a multiple linear regression analysis is attempted for predicting the tuned controller gains in accordance with the weather conditions. A linear relationship can be observed between the average temperature of each day and the *K_p_, K_i_*_,_ and *K_d_* gains (Figure 21).

Linear relationships can also be noticed between the average humidity and the controller gains, as well as average wind speed and the controller gains. On the other hand, no linear relationship was identified between the average atmospheric pressure and the controller gains. Thus, the average atmospheric pressure is removed from the analysis. The results of the multiple linear regression are presented in Table 4.

The Multiple R value is greater than 0.9 for all predicted controller gains, emphasizing a high degree of linear relationship among the variables. On the other hand, the coefficient of determination (R Square) has a minimum value of 0.82 for *K_p_*, meaning that 82% variation in the dependent variable can be explained by the independent one. The adjusted R Square value has a minimum value of 0.80 for *K_p_*, confirming the fact that 80% of the points fit the regression line. Overall, the precision of the multiple regression analysis is characterized by a maximum standard error of 1.49% in case of the *K_d_* gains.

Based on the general form of the regression analysis [53]:

(34) $$Y=\beta_{0}+\beta_{1}X_{1}+\beta_{2}X_{2}+\beta_{3}X_{3}.$$

The intercept (β_0_) and each corresponding coefficients (β_1…3_) are presented in Table 5 for predicting the *K_p_*, *K_i_*, or *K_d_* gains (*Y*) by knowing the average temperature (*X*_1_), average humidity (*X*_2_), and average wind speed (*X*_3_) for a given day.

### 3.9. LSTM Generalization

The next objective of the present work consists of generalizing the behavior of the tuned control methodology for any given weather conditions. In this way, the approach can be implemented successfully as part of smart home automation systems.

**Figure 22:**
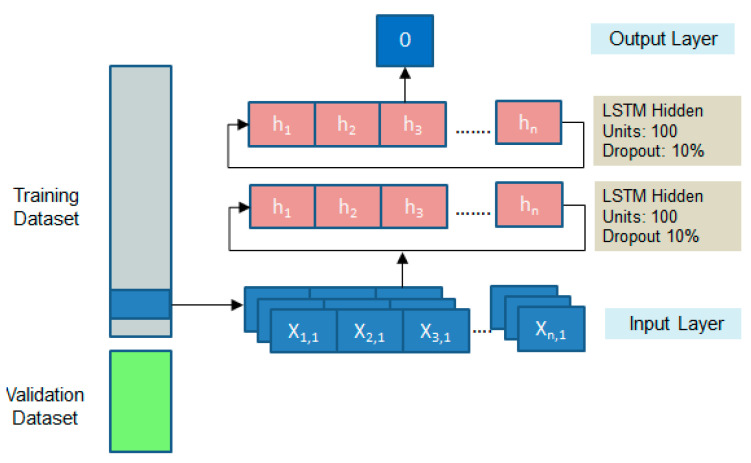
Graphical representation of the LSTM and its hyperparameters.

The LSTM recurrent neural network is employed to generalize the behavior of the actuating signal based on the weather conditions and interior temperature. In the proposed model, the input layer consists of a 3D array comprising sequential data regarding the ambient temperature (°C), humidity (%), wind speed (Km/h), and interior temperature (°C). All features were normalized by employing the min–max scaling technique. A stack of two LSTM layers is added to capture the abstract concepts in the sequences. The rectified linear unit is used as activation function. The generalization ability of the model is improved by employing the dropout regularization method. Thus, LSTM units are randomly excluded during the training process. The choice of hidden units and dropout rates that ensure an optimal convergence were decided as: 100 hidden units for both layers, having a dropout rate of 10% (Figure 22).

In the output layer, a continuous value is predicted that corresponds to the behavior of the actuating signal. The iterative update of the weights is achieved by employing the Adam optimizer. Typical to a regression problem, the mean squared error is used for evaluating the convergence of the model.

The response of the thermostat is recreated by considering a wide range of operational scenarios in the Simulink–SAMCEF environment:

1. Ideal conditions occur when the weather is found in an equilibrium state for long time periods. In this scenario, the effort of the controller to maintain the set point value is low;
2. Step response occurs due to the sudden change of the weather conditions (i.e., varying ambient temperature or wind speeds). This state is characterized by spikes in the actuating signal that compensate the disturbances;
3. Limit response is characterized by severe weather conditions (i.e., days with average high wind speeds and low temperatures). In this operational state, the actuating signal is close to its saturation limit;
4. Limit overshot represents an operational state in which the weather conditions cause the controller to switch off (i.e., ambient temperatures that exceed the set point value). During such periods, the actuating signal decreases to 0 while overshoot from the set point occurs due to the external factors.

The aim of the simulation study is not to accurately recreate weather conditions, but rather to provide examples of the actuating signal behavior under different cases of disturbances for facilitating the supervised learning process. In total, 90,000 rows are provided for training and test purposes.

Of the values, 70% are used for training the model and the remaining 30% are used for evaluating the prediction accuracy. TensorFlow 2.0 with Keras machine learning library is employed for defining the LSTM architecture. A total number of 5 epochs is chosen for training. During each epoch, the mean square error loss function is monitored for evaluating the prediction accuracy of the model. After the first epoch, the loss function had a value of 0.0495 while at epoch 5 the value was minimized to 0.0060.

**Figure 23:**
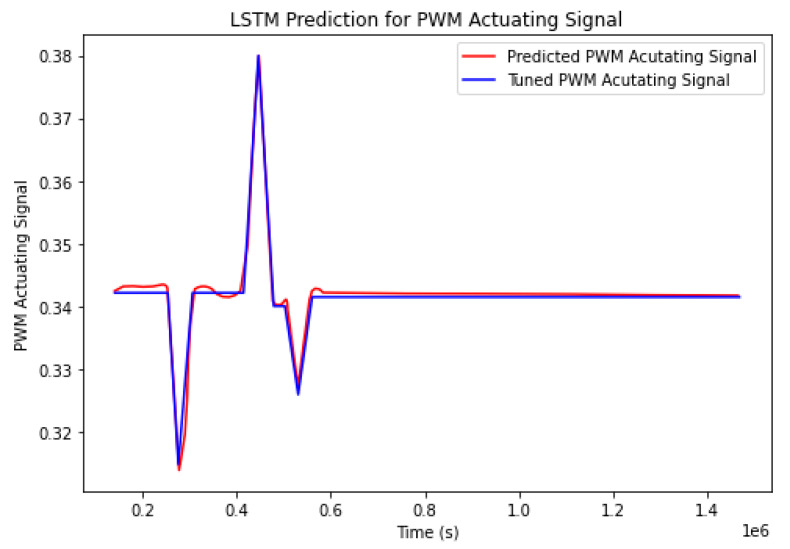
The response of the LSTM actuating signal in comparison with the tuned control methodology for the 18 November.

The accuracy of the model is tested by comparing the predicted actuating signal by the LSTM with the tuned control methodology for the 18 November. Figure 23 depicts the resulting curves.

The findings indicated that the actuating signal predicted by the LSTM neural network has the ability to recreate the behavior of the actuating signal that is generated by the tuned control methodology with high fidelity. The following error metrics confirm the accuracy of the model:

- Mean Absolute error: 0.0025;
- Mean Squared error: 1.60 · 10^−5^;
- Root Mean Squared Error: 0.004.

**Figure 24:**
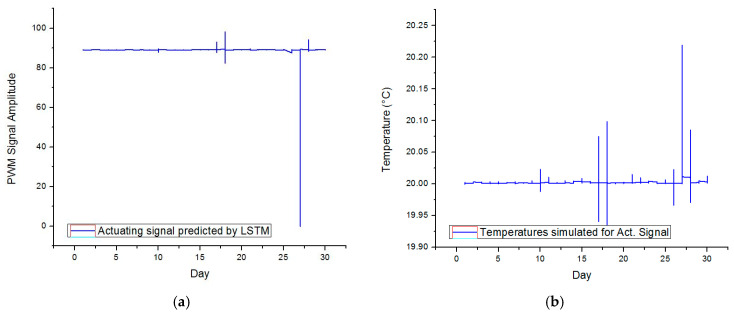
(**a**) The actuating signal predicted by LSTM and (**b**) the simulated temperatures.

The LSTM methodology is further employed for predicting the behavior of the tuned controller for November 2021.To this end, the inputs of the model consist of the weather condition and the temperatures recorded inside the enclosure (derived from the simulation model). The information is extracted for intervals of 30 min. Thus, the resulting test dataset includes a total of 1440 rows. Figure 24 depicts the shape of the predicted actuating signal and the temperatures achieved by simulation for the given inputs.

## 4. Results and Discussion

The power draw of the generalized controller can be estimated as 10.15 KWh. From this perspective, the proposed approach achieves 4% more power efficiency compared to the initial thermostat that was tuned in an ideal environment. Furthermore, the overshoot was minimized, given the maximum temperature error of +0.21 °C that occurred on the 27 November.

However, Figure 24 emphasizes localized situations when the actuating signal exceeds the lower saturation limit bounds (also on the 27 November). In this case, the PWM signal has a negative value of –0.15 that cannot occur in practice. Changes are required to the proposed algorithm for filtering such values.

The reliability of the study and outcomes from the simulation model are verified in three stages:

1. By matching the conductance and capacitance of the lumped parameter model with the experimental data. In this regard, the temperatures derived from the simulation model match the temperature curves derived from the experiments under step-loading;
2. By testing the control methodology that was developed with the support of the simulation model on a physical thermostat circuit. In this regard, the ability of the PID controller to maintain its setpoint value proves the accuracy of the simulated vs. real-world plant;
3. By generalizing the behavior of the actuating signal that was derived by the improved control methodology. The stable operation and increased power efficiency of the thermostat confirms the validity of the given concepts.

A wide amount of literature on the thermal modeling of residential buildings is available. The described approaches rely less on experimental data in the development stage, as compared to this work. However, they involve high computation demands because they rely on computational fluid dynamics software. Thus, their use in conjunction with numerical computing environments is limited. In comparison, the approach presented in this paper can be employed for extended simulation studies given the efficiency of the reduced order model. Even so, computational fluid dynamics software is employed most of the time for explicitly modeling the fluid flow and its impact on the thermal behavior of the living space. From this perspective, the existing methodologies take into account the 3D representation of both solid and fluid domains of the problem. Therefore, the computational demands of such methodologies are high. Furthermore, their use in conjunction with numerical computing environments is limited.

**Limitations of the work**: As seen in the previous section, the LSTM model does not fully take into account the lower saturation bounds. Another important limitation of the research is the fact that the experimental setup does not fully recreate the heat transfer that occurs in residential buildings, given the living habits of the occupants.

**Future work**: Future work will focus on developing an algorithm that can improve the LSTM predictions by filtering the actuating signal values that exceed the saturation limits of the actuator. Moreover, the neural model can be modified to include the behavioral patterns of occupants in the learning process. Thus, the approach can be correlated directly with a smart home automation system.

## 5. Conclusions

The present paper proposes a new approach for estimating the heat transfer in residential buildings by employing a lumped parameter simulation model. Both the fluid and the solid domains of the problem are captured by means of 2D membrane elements. A laboratory scale experimental setup is developed, comprising common building and insulation materials. The thermal behavior of the interior space is evaluated under step-load heating in a disturbance free environment. Given the first-order representation of the process, thermal contact conductance and capacitance are included at the fluid domain levels for replicating the temperature gain and time constants of the experimentally derived temperature curves. The results achieved confirm the ability of the lumped parameter simulation model to recreate the heat transfer that occurs in real-world conditions. Coupling between MATLAB Simulink and the SAMCEF solver is carried out for developing and tuning a PID thermostat. The experimental setup is tested in exterior conditions for one month. Changes in the behavior of the plant occur due to the varying weather. Therefore, superior controller efficiency can be achieved for each calendar day by retuning the controller gains. A multiple linear regression analysis is developed, considering the average temperature, wind speed, and humidity for one day as independent variables, and the tuned controller gains as the dependent ones. The improved methodology was verified for the 18 November, given the high process disturbances of that date. Compared to the initial controller, the proposed approach achieved 4% more energy efficiency while minimizing the temperature error from +0.8 °C to +0.25 °C. In the next stage, multiple operational scenarios were simulated in the Simulink–SAMCEF environment. The results were used to train an LSTM recurrent neural network. In this way, the actuating signal of the improved controller can be predicted in accordance with the weather data and the interior temperature.

## Figures and Tables

**Table 1:** Detailed specifications of the elements of the laboratory scale test platform (Figure 7).

Number	Name of the Component	Description
1	Side walls polystyrene insulation	15 mm thickness expanded polystyrene insulation panels
2	Plexiglas window	100 × 100 × 3 mm Plexiglas panel
3	Poplar plywood celling/floor	6 mm thickness
4	Poplar plywood walls	
5	Steel elbow	2 mm thickness standard steel
6	Group of resistors	30 W total power dissipation
7	PVC cornice	0.5 mm thickness
8	Tc-k thermocouple	±0.25 °C resolution

**Table 2:** Data regarding weather conditions for the 10 and 18 November 2021.

Date	Temperature (°C)			Humidity (%)			Wind Speed (Km/h)			Pressure (mmHg)		
	Min	Max	Avg.	Min	Max	Avg.	Min	Max	Avg.	Min	Max	Avg.
10 November 2021	0	8.88	4.36	53	100	81.1	1.6	14.4	7.3	768	771	770
18 November 2021	1.11	8.9	5.56	54	93	74.3	0	11.2	6.23	759.7	763.5	760.8

**Table 3:** A selection of different *TCC* and *C_th_* corrections.

Day	Average Temperature (°C)	Average Humidity (%)	Average Wind Speed (Km/h)	Average Pressure (mmHg)	Corrections		Tuned Gains		
					*TCC* (%)	*C_th_* (%)	*K_p_*	*K_i_*	*K_d_*
1 November 2021	10.04	85.4	3.97	753.8	−4.23	+5.51	26.393	0.178	−228.965
5 November 2021	13.73	78.6	3.52	753.9	+0.08	+2.34	24.485	0.161	−219.555
10 November 2021	4.36	81.1	7.3	770	−6.2	+7.7	27.489	0.190	−233.453
13 November 2021	5.23	88.8	7.51	759	−7.00	+7.59	27.719	0.191	−235.467
18 November 2021	5.56	74.3	6.23	760.8	−4.97	+6.23	26.775	0.182	−230.648
24 November 2021	3.00	86.3	4.76	762.1	−6.7	+6.81	27.413	0.188	−234.830
26 November 2021	2.99	85.3	5.86	748.4	−7.55	+6.68	27.642	0.189	−236.997
29 November 2021	7.88	89.18	11.7	738.5	−8.95	+9.12	28.713	0.201	−240.261
30 November 2021	3.13	86.7	11.7	744.7	−10.6	+10.1	29.504	0.208	−244.545

**Table 4:** Results of the multiple linear regression analysis.

Regression Statistics			
	Prediction for Kp	Prediction for Ki	Prediction for Kd
**Multiple R**	0.910	0.913	0.964
**R Square**	0.828	0.834	0.930
**Adjusted R Square**	0.809	0.815	0.922
**Standard Error**	0.532	0.005	1.493
**Observations**	30		

**Table 5:** Intercept and coefficients of the multiple linear regression analysis.

Intercept and Coefficients			
	Prediction for Kp	Prediction for Ki	Prediction for Kd
**Intercept**	20.924	0.136	−194.030
**Average temperature (°C)**	−0.150	−0.001	0.917
**Average humidity (%)**	0.069	0.001	−0.431
**Average wind speed (Km/h)**	0.296	0.003	−1.107

## Data Availability

Data used in this study is available on request from the first author.
